# Circulating Long Non-Coding RNA GAS5 Is Overexpressed in Serum from Osteoporotic Patients and Is Associated with Increased Risk of Bone Fragility

**DOI:** 10.3390/ijms21186930

**Published:** 2020-09-21

**Authors:** Virginia Veronica Visconti, Simona Fittipaldi, Simone Ciuffi, Francesca Marini, Giancarlo Isaia, Patrizia D’Amelio, Silvia Migliaccio, Claudio Marcocci, Salvatore Minisola, Ranuccio Nuti, Giuseppe Novelli, Maria Luisa Brandi, Annalisa Botta, Umberto Tarantino

**Affiliations:** 1 Department of Biomedicine and Prevention, Medical Genetics Section, University of Rome Tor Vergata, Via Montpellier 1, 00133 Rome, Italy; virginia.veronica.visconti@uniroma2.it (V.V.V.); simona.fittipaldi@uniroma2.it (S.F.); novelli@med.uniroma2.it (G.N.); 2 Department of Orthopedics and Traumatology, PTV Foundation, 00133 Rome, Italy; umberto.tarantino@uniroma2.it; 3 Department of Experimental and Clinical Biomedical Sciences Mario Serio, University of Study of Florence, Viale Pieraccini 6, 50139 Florence, Italy; simone.ciuffi@unifi.it (S.C.); f.marini@dmi.unifi.it (F.M.); marialuisa.brandi@unifi.it (M.L.B.); 4 Department of Medical Science, Gerontology Section, University of Turin, Corso Bramante 88/90, 10126 Turin, Italy; giancarlo.isaia@unito.it (G.I.); patrizia.damelio@unito.it (P.D.); 5 Geriatric Unit, Centre Hospitalier Universitaire Vaudois (CHUV), University of Lausanne, 1011 Lausanne, Switzerland; 6 Department of Movement, Human and Health Sciences, University of Foro Italico of Rome, 00135 Rome, Italy; silvia.migliaccio@uniroma4.it; 7 Department of Clinical and Experimental Medicine, Endocrinology Unit II, University of Pisa and University Hospital of Pisa, Via Paradisa 2, 56124 Pisa, Italy; claudio.marcocci@med.unipi.it; 8 Department of Clinical, Internal, Anaesthesiologic and Cardiovascular Sciences, Sapienza, Rome University, Viale del Policlinico 155, 00161 Rome, Italy; salvatore.minisola@uniroma1.it; 9 Department of Medicine, Surgery and Neuroscience, University of Siena, Policlinico Le Scotte, Viale Bracci 2, 53100 Siena, Italy; nutir@unisi.it; 10 IRCCS NEUROMED, 86077 Pozzilli, IS, Italy; 11 Department of Pharmacology, School of Medicine, University of Nevada, Reno, NV 89557, USA; 12 Unit of Bone and Mineral Diseases, University Hospital of Florence, Largo Palagi 1, 50139 Florence, Italy; 13 Department of Clinical Sciences and Translational Medicine, University of Rome Tor Vergata Rome, 00133 Rome, Italy

**Keywords:** osteoporosis, biomarker, epigenetics, lncRNA GAS5, bone fragility

## Abstract

Osteoporosis (OP) is a multifactorial disorder in which environmental factors along with genetic variants and epigenetic mechanisms have been implicated. Long non-coding RNAs (lncRNAs) have recently emerged as important regulators of bone metabolism and OP aetiology. In this study, we analyzed the expression level and the genetic association of lncRNA GAS5 in OP patients compared to controls. Quantitative RT-PCR analysis of GAS5 was performed on the serum of 56 OP patients and 28 healthy individuals. OP subjects were divided into three groups of analysis: 29 with fragility fractures of lumbar spine (OP_VF), 14 with fragility fractures of femoral neck (OP_FF) and 13 without fractures (OP_WF). Genotyping of the rs145204276 insertion/deletion polymorphism has also been performed by Restriction fragment length polymorphism (RFLP) and direct sequencing analyses. Expression of circulating GAS5 is significantly increased in OP patients compared to controls (*p* < 0.01), with a statistically higher significance in fractured OP individuals vs. healthy subjects (*p* < 0.001). No statistically significant change was found in female OP patients; conversely, GAS5 is upregulated in the subgroup of fractured OP women sera (*p* < 0.01) and in all OP males (*p* < 0.05). Furthermore, a direct correlation between GAS5 expression level and parathyroid hormone (PTH) concentration was found in OP patients (*r* = 0.2930; *p* = 0.0389). Genetic analysis of rs145204276 revealed that the deletion allele was correlated with a higher expression of GAS5 in OP patients (0.22 ± 0.02 vs. 0.15 ± 0.01, ** *p* < 0.01). Our results suggest circulating GAS5 as a putative biomarker for the diagnosis and prognosis of OP and OP-related fractures.

## 1. Introduction

**Figure 1:**
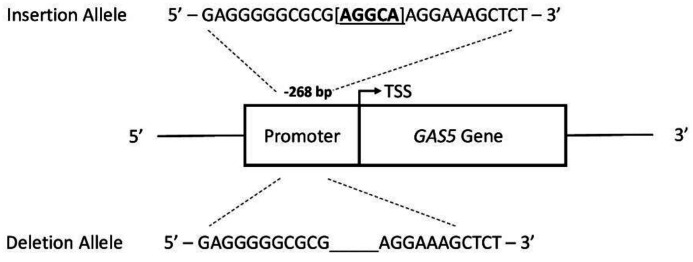
Schematic representation of the rs145204276 located in the promoter region of *GAS5* gene.

Osteoporosis (OP, MIM: #166710) is a skeletal disorder characterized by compromized bone strength, low bone mineral density (BMD) and an increased risk of fracture and bone fragility [1]. At the cellular level, OP is linked to an imbalance between the resorption of old bone by osteoclasts and its subsequent replacement by osteoblasts leading to alterations in bone microarchitecture, resulting in a higher risk of spontaneous bone fractures [2]. During recent years, many epigenetic mechanisms have been correlated with OP pathogenesis [3]—these include DNA methylation, post-translational modifications of histone tails and non-coding RNAs (ncRNAs) [4]. In particular, a class of ncRNAs called long non-coding RNAs (lncRNAs) plays a relevant role in the epigenetic regulation of gene expression [5]. lncRNAs are a class of regulatory ncRNAs ranging in size from >200 nt to 100 kb that cannot code for proteins. Despite their recent discovery, different studies demonstrate that lncRNAs are involved in a wide range of physiological and pathological processes as they act at different levels of gene regulation by interacting directly with DNA, RNA, and proteins [6]. lncRNAs are important regulators of bone metabolism and changes in their expression pattern are linked to OP pathogenesis [7,8]. Recently, we demonstrated that lncRNAs involved in inflammatory responses are deregulated in cells from OP patients compared to controls [9]. Among them, the lncRNA *growth arrest-specific transcript 5* (GAS5, MIM: #608280), encoded by the *GAS5* gene at 1q25, has shown a five-fold downregulation of OP primary osteoblasts, thus indicating a possible role in OP [9]. GAS5 has been widely studied in the field of cancer research where it acts as tumor suppressor by regulating cell cycle arrest and cell death [10,11,12,13,14]. Moreover, genetic analysis of GAS5 has led to the identification of a functional 5-base pair (AGGCA/-) insertion/deletion (indel) polymorphism, rs145204276, located in the promoter region 268-bp upstream of the transcription start site (TSS) (Figure 1). This polymorphism is capable of modulating Gas5 expression level and has been associated with the susceptibility to several cancer types [15,16,17]. GAS5 has been demonstrated to regulate inflammation, proliferation and apoptosis in different pathological conditions [14,18,19]. Although the actions of GAS5 are not completely elucidated, some mechanisms have been proposed [13,20]. In particular, a direct interaction of GAS5 with the DNA binding domain of the glucocorticoid receptor blocking steroid hormone receptor has been described [18]. Moreover, GAS5 can function as a competing endogenous RNA (ceRNA) to regulate microRNAs’ signalling pathways and functions [20]. For instance, GAS5 is predicted to inhibit miR-21, which also regulates osteoclast formation [14], and, in turn, miR-21 is capable of suppressing GAS5 expression in a reciprocal feedback loop [21,22]. Besides, Wang and colleagues have observed that GAS5 overexpression in nucleus pulpus cells repressed Bcl-2 and upregulated caspase-3, suggesting lncRNA GAS5 is a potential therapeutic target for intervertebral disc degeneration [19]. Recently, GAS5 deregulation was also found in human multipotential mesenchymal stem cells (hMSCs) derived from OP patients and in serum from postmenopausal osteoporotic women [23,24]. In this study, we determined the levels of circulating GAS5 in serum from a cohort of OP patients (*n* = 56) compared to control individuals (*n* = 28). Moreover, we also studied the functional correlation of the GAS5 rs145204276 polymorphism and its expression level in OP and control groups.

## 2. Results

### 2.1. Clinical Characteristics of Individuals Included in the Study

The clinical characteristics of the study samples (OP, *n* = 56; controls CTRs; *n* = 28) are presented in Table 1. OP subjects were divided into two groups: OP patients with fragility fractures (OP_F; *n* = 43), which included the subgroups of patients presenting vertebral (OP_VF; *n* = 29) or femoral (OP_FF; *n* = 14) fractures, and OP patients without fractures (OP_WF; *n* = 13). The average age was 68.2 ± 4.9 years for OP patients and 67 ± 4.9 years for healthy controls. OP group patients were characterized by a *t*-score ≤−2.5 and healthy controls by a *t*-score ≥ −1.0. The mean body mass index (BMI) was significantly higher in the OP group compared to CTRs (28.7 ± 5.7 vs, 24.4 ± 3.3 *p* < 0.001). In addition, there was no statistically significant difference in clinical characteristics between OP and CTR groups, except for the mean circulating alkaline phosphatase (ALP; U/l) and serum C-terminal telopeptide (s-CTX; ng/mL), which were higher in OP patients compared to CTRs (91 ± 37 vs. 71 ± 15, *p* = 0.0096 and 1.65 ± 2.80 vs. 3.45 ± 4.20, *p* = 0.0013, respectively) (Table 1).

### 2.2. GAS5 Expression Level Is Significantly Upregulated in Serum from OP Patients with Fragility Fractures 

**Figure 2:**
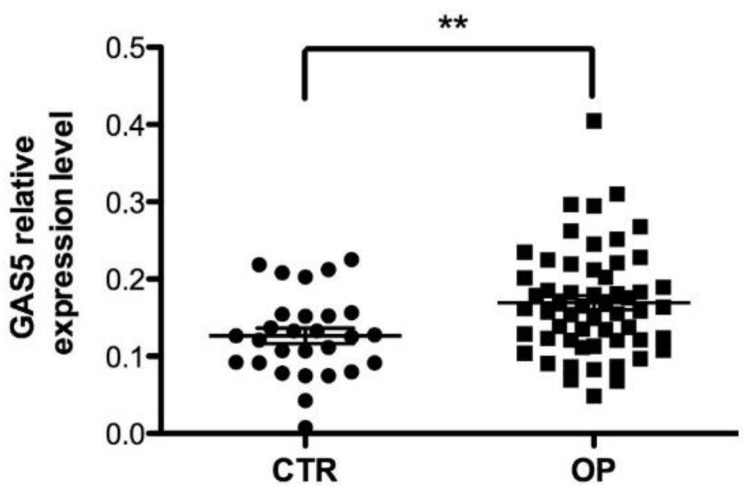
Serum expression level of GAS5 in OP patients. *GAS5* expression level was analyzed in serum from 56 OP patients and 28 healthy controls. GAPDH mRNA level was used to normalize the relative amount of GAS5 and relative expression values are expressed as 2^−ΔCt^. Individual data points represent the mean of duplicate assays for each sample. Statistical differences between OP and CTR groups were analyzed by non-parametric Mann–Whitney *U*-test. Error bars indicate mean ± standard error mean (s.e.m.) ** *p* < 0.01.

GAS5 expression levels were analyzed by qRT-PCR in serum samples from 56 OP patients (14 males and 42 females) and 28 CTRs (8 males and 20 females). Our results demonstrated that the mean levels of circulating GAS5 in OP patients was higher than that of CTR individuals (Figure 2; *p* = 0.005). 

**Figure 3:**
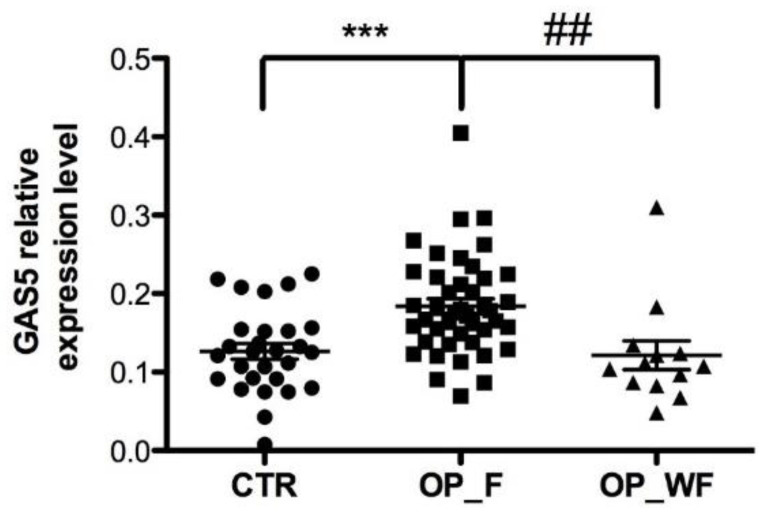
Serum expression level of GAS5 in OP patients with and without fragility fractures. The GAS5 expression level in the serum from 43 OP patients with fragility fractures (OP_F) was compared to 13 OP patients without fractures (OP_WF) and 28 healthy samples (CTR). The *GAPDH* mRNA level was used to normalize the relative amount of GAS5 and relative expression values are expressed as 2^−ΔCt^. Statistical differences between groups were analyzed using ANOVA with Tukey’s multiple comparison post-test analysis. Error bars indicate mean ± s.e.m. *** Represents a significant difference between OP_VF and CTR (*p* < 0.001); ## represents a significant difference between OP_VF and OP_FF (*p* < 0.01).

Next, in order to investigate a potential relationship between the expression level of GAS5 and occurrence of osteoporotic fractures, OP patients were divided into two groups with (*n* = 43) or without (*n* = 13) fragility fractures. We found a remarkably significant increase in the GAS5 expression level in OP_F compared to the CTR group and a significant increase in OP_F compared to OP_WF was also found (Figure 3; *p* < 0.001 and and *p* < 0.01, respectively). No difference between OP_WF and CTR groups was observed (Figure 3; *p* = 0.4).

**Figure 4:**
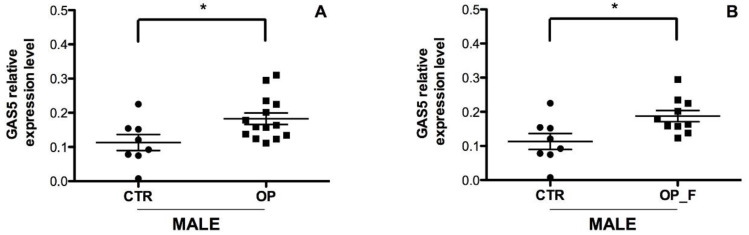
Serum expression level of GAS5 in OP male patients. (**A**) GAS5 expression level was analyzed in the serum of 14 OP total male, with and without fragility fractures, and 8 healthy male controls. * *p* < 0.05; (**B**) GAS5 expression level was analyzed in the serum of 10 OP fractured male and 8 healthy male controls. *GAPDH* mRNA level was used to normalize the relative amount of GAS5 and relative expression values are expressed as 2^−ΔCt^. Individual data points represent the mean of duplicate assays for each sample. Statistical differences between groups were analyzed for significance using unpaired Student’s t-test. Error bars indicate mean ± s.e.m. * *p* < 0.05.

Of note, OP patients with vertebral fractures (OP_VF; *n* = 29) showed the most relevant increase in GAS5 expression level compared to both CTR and OP with femoral fractures (OP_FF; *n* = 14) (*p* < 0.0001; Figure S1). Subsequently, to investigate a possible gender-dependent effect on GAS5 expression level, we analyzed OP male and female patients separately and found a significant increase in GAS5 in the OP male group compared to the CTR male group (Figure 4A,B; *p* = 0.02).

**Figure 5:**
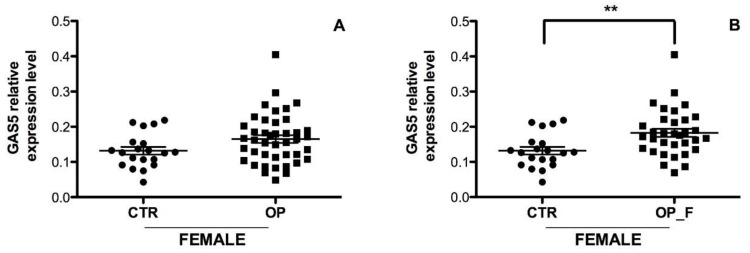
Serum expression level of GAS5 in OP female patients. (**A**) GAS5 expression level was analyzed from serum of 42 OP females, with and without fragility fractures, and 20 healthy females. NS; (**B**) GAS5 expression level was analyzed in the serum from 33 OP fractured females and 20 healthy females. *GAPDH* mRNA level was used to normalize the relative amount of GAS5 and relative expression values are expressed as 2^−ΔCt^. Individual data points represent the mean of duplicate assays for each sample. Statistical differences between groups were analyzed for significance using unpaired Student’s *t*-test. Error bars indicate mean ± s.e.m. ** *p* < 0.01.

Conversely, no significant difference was found in the GAS5 expression level between OP females (Figure 5A) and CTR females. However, when only females patients with fractures (OP_F = 33) were considered, GAS5 was significantly increased (Figure 5B, *p* = 0.005) compared to CTR females, suggesting that an increase in GAS5 expression could be associated with the presence of fragility fractures.

### 2.3. Correlation between Serum Circulating GAS5 with Clinicopathological Characteristics of Osteoporotic Patients

After adjusting for age and BMI, the relationship between the expression levels of GAS5 and clinicopathological characteristics of patients—including t-scores (femoral neck, FN and lumbar spine L1/L4), calcium, phosphorus, 25-hydroxyvitamin D (25-(OH)-Vit D), ALP, bone alkaline phosphatase (BALP), s-CTX and parathyroid hormone (PTH) concentrations—was studied using Spearman’s correlation coefficient. This analysis showed only a positive significant correlation between the expression levels of GAS5 and PTH concentrations within the group of OP patients (r = 0.2930; *p* = 0.038; Figure S2). Moreover, as expected, a negative and significant correlation was also found between PTH and 25-(OH)-VitD concentrations in serum from OP patients (r = −0.4627; *p*= 0.0007; Figure S3). There were no significant correlations between GAS5 expression levels and all the other biochemical parameter analyzed.

### 2.4. The rs145204276 Polymorphism Is Associated with the Expression Levels of GAS5 

We first determined the frequencies of Ins/Ins, Ins/Del and Del/Del genotypes in our study samples. Table 2 summarizes alleles’ and genotypes’ distribution of rs145204276 in OP patients and CTRs.

**Figure 6:**
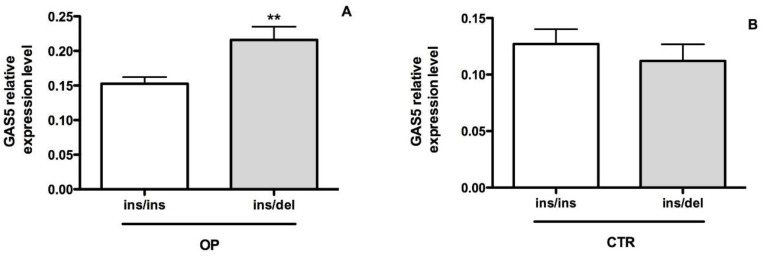
Expression level of GAS5 in OP and CTR individuals with different rs145204276 genotypes (**A**) GAS5 expression in OP patients with Ins/Del (*n* = 17) and Ins/Ins (*n* = 39) genotypes (0.22 ± 0.02 vs. 0.15 ± 0.01, ** *p* < 0.01). (**B**) GAS5 expression CTR subjects with genotype Ins/Del (*n* = 7) and Ins/Ins (*n* = 21), respectively (0.12 ± 0.05 vs. 0.11 ± 0.03 *p* = 0.5). Statistical differences between groups were analyzed for significance using unpaired Student’s *t*-test. Error bars indicate mean ± s.e.m.

The rs145204276 allelic frequencies were: Ins = 84.8% and Del = 15.2% in OP patients and Ins = 87.5% and Del = 12.5% in CTRs. These allelic frequencies, even if determined in a small sample size, fully agreed with the allelic frequencies of the rs145204276 polymorphism reported in the 1.000 Genomes Project database (https://www.internationalgenome.org/). The distribution of rs145204276 genotypes was as follows: heterozygotes Ins/Del (*n* = 17) and homozygotes Ins/Ins (*n* = 39) in OP patients; heterozygotes Ins/Del (*n* = 7) and homozygotes Ins/Ins (*n* = 21) in CTRs. No Del/Del genotypes were detected in our study sample. No significant differences between OP and CTRs in the rs145204276 allelic (*p* = 0.64) and genotypic (*p* = 0.61) frequencies were found. Considering the allelic distribution, the OR is 1.25 with 95% confidence limits = 0.48 and 3.22 (Table 2). Deviations from the Hardy–Weinberg equilibrium were not observed for rs145204276 in OP patients, indicating that this polymorphism is not associated with the susceptibility to OP in the analyzed patients. In order to test if the rs145204276 polymorphism could have a functional effect on the expression of lncRNA GAS5, we further examined the level of circulating GAS5 in OP patients and CTRs with different genotypes (Figure 6).

OP patients with Ins/Del genotype (*n* = 17) revealed a higher expression of GAS5 than those with Ins/Ins genotype (*n* = 39) (0.22 ± 0.02 vs. 0.15 ± 0.01, ** *p* < 0.01). Conversely, CTR subjects with Ins/Ins genotype (*n* = 21) showed no difference in GAS5 expression as compared with those with Ins/Del genotype (*n* = 7) (0.12 ± 0.05 vs. 0.11 ± 0.03 *p* = 0.5).

## 3. Discussion

Recent in vitro and in vivo studies have suggested that GAS5 is implicated in the function of bone cells and potentially in OP pathogenesis. Our study further confirms and extends this hypothesis demonstrating that the circulating GAS5 level is significantly increased in OP patients, specifically in subjects presenting fragility fractures. GAS5 expression level was found downregulated in hMSCs [24] and primary osteoblasts [9] from OP patients and its overexpression significantly induced ALP activity and promoted osteogenic differentiation of hMSCs through RUNX2 induction [24]. Moreover, Wang et al., by using an osteoporotic-induced mouse model, observed that GAS5 expression was increased during the osteogenic differentiation of bone marrow mesenchymal stem cells (BMSCs) via negatively modulating miR-135a-5p to induce FOX1 expression [25]. Our results would appear to be in conflict with the above-mentioned studies which reported an upregulation of GAS5 linked to bone impairment. Nevertheless, these discrepancies could be explained with evidence indicating that lncRNAs can travel to neighbouring and distant cells in association with exosomes in response to specific signals [26,27,28,29]. It has been previously suggested that lncRNAs with low expression levels in cells could be enriched in secreted exosomes and transferred from cell to cell, presumably with a secretion mechanism [26,28]. Indeed, we speculate that an increase in circulating GAS5 in fractured OP patients could be explained with a mechanism in which the GAS5 level is induced from a proapoptotic signal to accumulate in exosomes, leading to a reduced expression level within osteoblasts and hMSCs. Notably, Koldemir and colleagues have demonstrated that GAS5 accumulates in exosomes in response to proapoptotic signals in breast carcinoma cells hypothesizing that this transcript could be involved in the communication of tumour cells upon cell death-promoting signals [27]. Besides, our results are corroborated by a previous study reporting a significant upregulation of circulating GAS5 expression levels in postmenopausal osteoporotic women with vertebral fractures [23]. An important target of GAS5 sponge action is *miR-21* and the GAS5/*miR-21* axis, which has been recognized to regulate proliferation and apoptotic pathways [14,21,30]. It is known that *miR-21* plays a key role in promoting BMSCs differentiation in osteogenic cells [31,32]. Moreover, a significant decrease in *miR-21* expression levels was found in plasma from OP postmenopausal women [33] and serum sample from OP patients [32] compared to healthy controls. Interestingly, *miR-21* serum levels were significantly lower in OP patients who had sustained vertebral fractures compared with those without fractures [7]. Vertebral fractures are the most prevalent type of osteoporotic fractures in postmenopausal women and the spine fracture status represents the most important risk factor for future fracture [34]. A recent study indicates that GAS5 upregulation is related to intervertebral disc degeneration due to its proapoptotic effects [19]. We also found the most significant GAS5 upregulation in OP_VF with respect to OP_FF. Although our study does not elucidate the mechanism of GAS5, it is possible to speculate that the upregulation of GAS5 is related to the decreased *miR-21* expression triggered by apoptotic pathways in remodelling processes occurring after bone fractures. Genotyping of the rs145204276 indel polymorphism within the GAS5 promoter correlated the Ins/Del genotype in OP patients with higher levels of GAS5 expression. Our results confirm previous studies indicating that the rs145204276 Del allele could lead to the increase in GAS5 transcription activity by epigenetic mechanisms [16,35]. Interestingly, we were unable to find a similar correlation in the CTR group, but this result must be considered preliminary because of the small sample size of the CTR compared to OP groups and deserves further investigations. We also found a direct correlation between GAS5 expression and PTH levels and an inverse correlation between 25(OH)-VitD and PTH concentrations in OP sera, confirming that low 25(OH)-VitD levels induce an increased PTH secretion to maintain calcium homeostasis [36,37]. We recognize that our study has some limitations. First, a higher number of women (*n* = 62) compared to men (*n* = 22) were analyzed. In addition, there was a significantly higher number of females presenting fractures compared to patient without fractures within the OP group. However, it is important to note that highly age-homogenous groups (60–75 years) were included in this study. Therefore, since OP, as well as the rate of osteoporotic fractures, are more frequent in postmenopausal women than age-matched men [38], a gender imbalance is a common feature in research studies in this field. Although our gender-specific stratified analysis suggests an association between GAS5 overexpression and the presence of fractures in OP females, the paucity of OP males with respect to OP female patients may not have the statistical power to show a gender-specific effect in fractured OP. Further studies on larger cohorts are required to address the significance of GAS5 deregulation in OP and its putative targets (e.g., *miR-21*) related to osteoporotic fractures.

## 4. Materials and Methods

### 4.1. Subjects

The study was approved by the Ethical Board of the Azienda Ospedaliero Universitaria Careggi (10419_oss; 1 March 2017); informed consent was obtained from all the participants. All experimental procedures were carried out according to The Code of Ethics of the World Medical Association (Declaration of Helsinki). All the collected data (clinical and genetic) were made appropriately anonymous, and each individual was identified, during the study, only by a unique alphanumeric identification code. Data were also analyzed as aggregates. This study included fifty-six patients affected by OP (14 males and 42 females) and twenty-eight healthy controls (CTRs; 8 males and 20 females) selected from a cohort of patients recruited from the following Italian clinical centers: “Umberto I” University Hospital, Rome; “AOU-Careggi”, Florence; “Tor Vergata” University Hospital, Rome; “AOU-City of Health and Science”, Turin; “Le Scotte” University Hospital, Siena; “Endocrine Unit 2” University Hospital, Pisa. Dual-energy X-ray absorptiometry (DEXA) evaluations were all performed within at most 24 months before the recruitment in this study and the collection of blood samples. All recruited subjects were aged between 60 and 75 years. Individuals affected by malignancies, endocrine disorders affecting bone and mineral metabolism, autoimmune diseases and bone disorders other than primary osteoporosis were excluded from the study, as well as those who underwent long-term therapy with drugs interfering with bone metabolism, sex hormone replacement therapy and/or antifracture and/or osteoanabolic therapies.

### 4.2. Specimen Collection

Whole blood from participants was drawn after overnight fasting and collected in tubes without anticoagulant. An aliquot of whole blood was stored at −80 °C for DNA extraction and genotyping analysis. To collect serum, each blood sample was centrifuged, within an hour of collection, at 1500× *g* for 20 min at 4 °C. Serum phase was harvested and transferred in RNase-free tubes. Additional centrifugation at 16,000× *g* for 10 min at 4 °C was performed, to remove residual cells/platelets/cell debris. Serum samples were frozen in aliquots and stored at −80 °C until further processing. 

### 4.3. Clinical and Biochemical Parameters 

According to the World Health Organization, densitometric diagnosis of OP based on technical DEXA evaluation of mineral density was carried out in every subject. Lumbar spine (L1-L4) and femoral (neck and total) scans were performed, and BMD was measured according to the manufacturer’s recommendation. The unit of measurement is represented by SD from the mean bone mass peak (T-score). All individuals were also subjected to the dosage of bone metabolism markers: 25-(OH)-Vit D, PTH, BALP, s-CTX, calcium and phosphorus levels were measured in fasting venous blood samples collected from the subjects. Finally, we collected data regarding age, gender, height, weight, dietary habit, smoke, alcohol intake, personal and familial clinical history. The detailed clinical characteristics of the study subjects are summarized in Table 1.

### 4.4. Haemolysis Evaluation

To avoid the negative effect of haemolysis on downstream processes, the levels of free haemoglobin in all serum samples were measured using a NanoDrop 1000 Spectrophotometer (Thermo Scientific, Waltham, MA, USA). Serum samples with an absorbance at 414 nm higher than 0.2 were considered to be haemolyzed and excluded from the subsequent analyses.

### 4.5. RNA Extraction and RT-qPCR Analysis of Circulating GAS5 Expression

Total RNA extraction was performed by the miRNeasy Serum/Plasma Advanced Kit (Qiagen, Hilden, Germany) according to the manufacturer’s instructions. For gene expression experiments, the reverse transcription (RT) and qPCR steps were conducted in the same reaction well using a power SYBR green RNA-to-Ct 1 step kit (TermoFisher Scientific, Waltham, USA) and the following cycles: 48 °C for 30 min, 95 °C for 10 min, followed by 95 °C for 15 s and 64 °C for 1 min for 40 cycles. The *GAPDH* gene was used as internal control (coefficient of variance (CV) percentage = 5.8%). PCR primers specific for *GAS5* and *GAPDH* genes were: GAS5 sense-5′-TGGTTCTGCTCCTGGTAACG-3′, antisense-5′-AGGATAACAGGTCTGCCTGC-3′; *GAPDH* sense-5′-GTCAAGGCTGAGAACGGGAA-3′, antisense-5′-AAATGAGCCCCAGCCTTCTC-3′. The comparative cycle threshold (Ct) method [2^−ΔCt^] [39] was used to analyze the relative GAS5 expression levels normalized to *GAPDH* levels as the internal control.

### 4.6. Rs145204276 Genotyping Assay

Genomic DNA was extracted from the peripheral blood of each subject using a FlexiGene DNA kit (Qiagen, Hilden, Germany), following the manufacturer’s instructions. The GAS5 promoter region containing the rs145204276 polymorphism was amplified with the following PCR pair primers: rs145204276_Fw (5′-TCCCGACTGAGGAGGAAGAGCA-3′) and rs145204276_Rv (5′- AACACCGTCCCGGAAGTGAAA-3′). Amplification was carried out in 35 µL volume, containing 75 ng genomic DNA, PCR reaction buffer (5×), 25 mM MgCl2, 1.25 mM dNTPs, 70pmol of each primer and 1U of Taq polymerase (Takara Bio, Mountain View, CA, USA). PCR conditions were the followings: 94 °C for 3 min, 30 cycles of denaturation at 94 °C for 40 s, annealing at 57 °C for 40 s and extension at 72 °C for 40 s. A final elongation was carried out at 72 °C for 10 min. Subsequently, PCR products were incubated for 7 h at 37 °C with a final inactivation step at 65 °C for 20 min with 5 U of BplI enzyme (ThermoFisher Scientific, Vilnius, Lithuania). The three different digestion patterns representative of the ins/ins, ins/del and del/del genotypes were resolved in 3% agarose gel. Genotypes at the rs145204276 locus were further confirmed by Sanger sequencing using an Applied Biosystems 3130xl Genetic Analyzer (ThermoFisher Scientific).

### 4.7. Statistical Analyses

Data were analyzed with GraphPad Prism 5.0 (GraphPad Software, Inc., La Jolla, CA, USA). Before using statistical test procedures, the assumptions of normality were verified for each variable applying the Shapiro–Wilk test. A non-parametric Mann–Whitney U-test was used for variables showing a skewed distribution whereas data following a normal distribution were processed with an unpaired Student’s t-test. The unpaired Student’s *t*-test was used to compare the expression of GAS5 among different genotypes of the rs145204276 polymorphism. A one-way ANOVA test, followed by pairwise comparisons using Tukey’s multiple comparison, was used to compare data from more than two groups. The correlation between qRT-PCR and clinical/biochemical data were evaluated using the Spearman’s rank test. Data are presented as the mean ± SE. Differences were considered significant when the *p* value was <0.05 (* *p* < 0.05, ** *p* < 0.01, *** *p* < 0.001).

## Figures and Tables

**Table 1:** Characteristics of Osteoporosis (OP) patients and healthy controls (CTRs).

Characteristics	OP (14 Males; 42 Females)	CTRs (8 Males; 20 Females)	*T*-test (Mann-Whitney Test)
Age (years)	68.16 ± 4.90	67.00 ± 4.95	NS (*p* = 0.304)
BMI (Kg/cm^2^)	24.38 ± 3.29	28.72 ± 5.70	*** (*p* < 0.001)
*t*-score L1-L4	−2.87 ± 1.09	0.23 ± 1.35	*** (*p* < 0.001)
*t*-score FN	−2.46 ± 0.82	0.58 ± 0.62	*** (*p* < 0.001)
Calcium (mg/dL)	9.21 ± 1.02	9.30 ± 0.48	NS (*p* = 0.675)
Phosphorus (mg/dL)	3.41 ± 0.65	3.28 ± 0.63	NS (*p* = 0.312)
PTH (pg/mL)	45.39 ± 24.66	45.29 ± 19.65	NS (*p* = 0.628)
25-(OH)-Vit D (ng/mL)	28.04 ± 11.01	27.02 ± 11.57	NS (*p* = 0.888)
ALP (U/L)	90.80 ± 36.63	70.77 ± 14.86	** (*p* = 0.0096)
BALP (μg/L)	16.67 ± 11.30	12.85 ± 4.80	NS (*p* = 0.169)
s-CTX (ng/mL)	3.45 ± 4.20	1.62 ± 2.80	** (*p* = 0.0013)

BMI, body mass index; PTH, parathyroid hormone; 25-(OH)-Vit D, 25-hydroxyvitamin D; ALP, alkaline phosphatase; BAPL, bone alkaline phosphatase; s-CTX, serum C-terminal telopeptide. ** (*p* < 0.01); *** (*p* < 0.001).

**Table 2:** Distribution of the genotypes and allelic frequencies of rs145204276 in OP patients and CTRs.

	Genotype			*p*	Allele		*p*	Odds Ratio (95% CI)
	Del/Del	Del/Ins	Ins/Ins		Del	Ins		
OP (*n* = 56)	0	17 (30.4%)	39 (69.6%)	0.61	17 (15.2%)	95 (84.8%)	0.64	1.25 (0.48–3.22)
CTRs (*n* = 28)	0	7 (25%)	21 (75%)		7 (12.5%)	49 (87.5%)		

## Supplementary Materials

Supplementary materials can be found at https://www.mdpi.com/1422-0067/21/18/6930/s1.

## Abbreviations

OP	Osteoporosis
CTR	Control
ncRNA	non-coding RNA
LncRNA	long non-coding RNA
OP_VF	OP vertebral fractures
OP_FF	OP femoral fractures
OP_WF	OP without fracture
BMD	bone mineral density
GAS5	growth arrest-specific transcript 5
ceRNA	competing endogenous RNA
hMSC	human multi-potential mesenchymal stem cell
DEXA	dual-energy x-ray absorptiometry
25-(OH)-Vit D	25-hydroxyvitamin D
PTH	parathyroid hormone
ALP	alkaline phosphatase
BALP	bone alkaline phosphatase
CTX	serum C-terminal telopeptide
BMI	body mass index
BMSC	bone marrow mesenchymal stem cell
