# Supplementary material for: Circulating Long Non-Coding RNA GAS5 Is Overexpressed in Serum from Osteoporotic Patients and Is Associated with Increased Risk of Bone Fragility
Source: Int J Mol Sci. 2020 Sep 21;21(18):6930. doi: 10.3390/ijms21186930 (PMC7554802; doi:10.3390/ijms21186930)
Supplement: Supplementary file 1 [file ijms-21-06930-s001.pdf]

Supplementary Figures Legends

Figure S1. Serum expression level of *GAS5* in OP patients with vertebral fractures (OP_VF) and femoral fractures (OP_FF). *GAS5* expression level in the serum of 29 OP_VF was compared to 14 OP_FF and 28 controls (CTR). *GAS5* expression level was normalized using *GAPDH* mRNA and relative expression values are expressed as 2-ΔCt. Individual data points represent the mean of duplicate assays for each sample. Statistical differences between groups were analysed using ANOVA with the Tukey's multiple comparison post-test analysis. *** represents a significant difference between OP_VF and CTR ($p<0.001$) ## represents a significant difference between OP_VF and OP_FF ($p<0.01$).

Figure S2. Correlation analysis of *GAS5* expression level and PTH concentration in OP patients serum. Level of *GAS5* expression was positively associated with PTH concentration (pg/ml). Spearman's correlation analysis was performed. Spearman r = 0.2930; 95% confidence interval = 0.007441 to 0.5344. *P < 0.05.

Figure S3. Correlation analysis of PTH and 25-(OH)-VitD concentrations in OP patients serum. Level of PTH (pg/ml) was negatively associated with PTH (pg/ml) concentration. Spearman's correlation analysis was performed. Spearman r = -0.3842; 95% confidence interval = -0.6040 to -0.1102. **P < 0.01.

Supplementary Figures

Figure S1

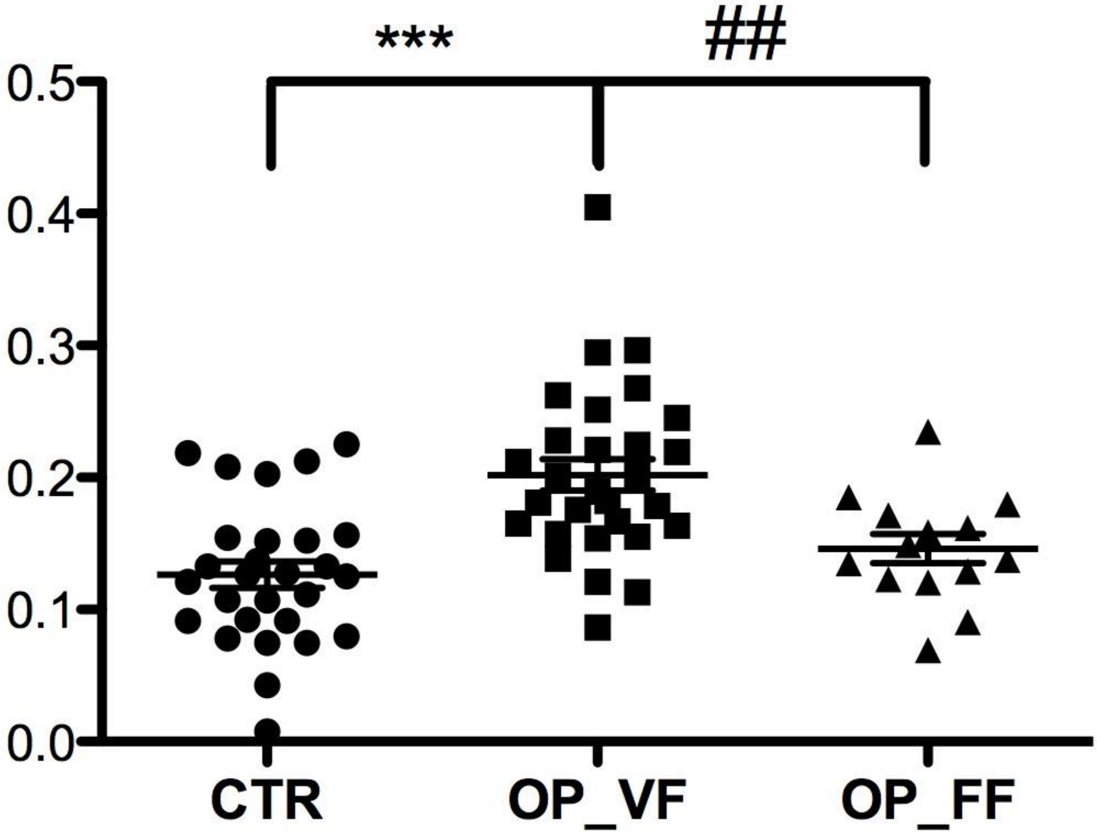

Figure S2

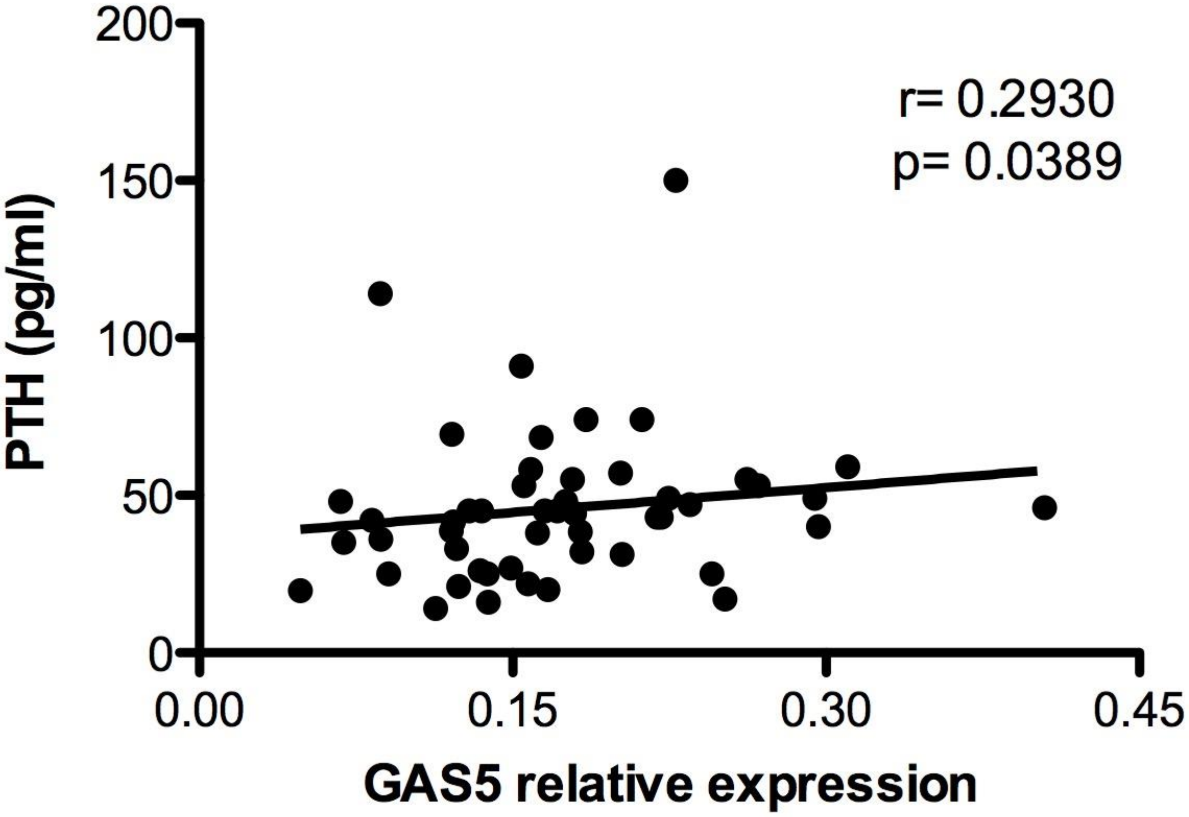
r= 0.2930
p= 0.0389
PTH (pg/ml)
GAS5 relative expression
200
150
100
50
0
0.00
0.15
0.30
0.45

Figure S3

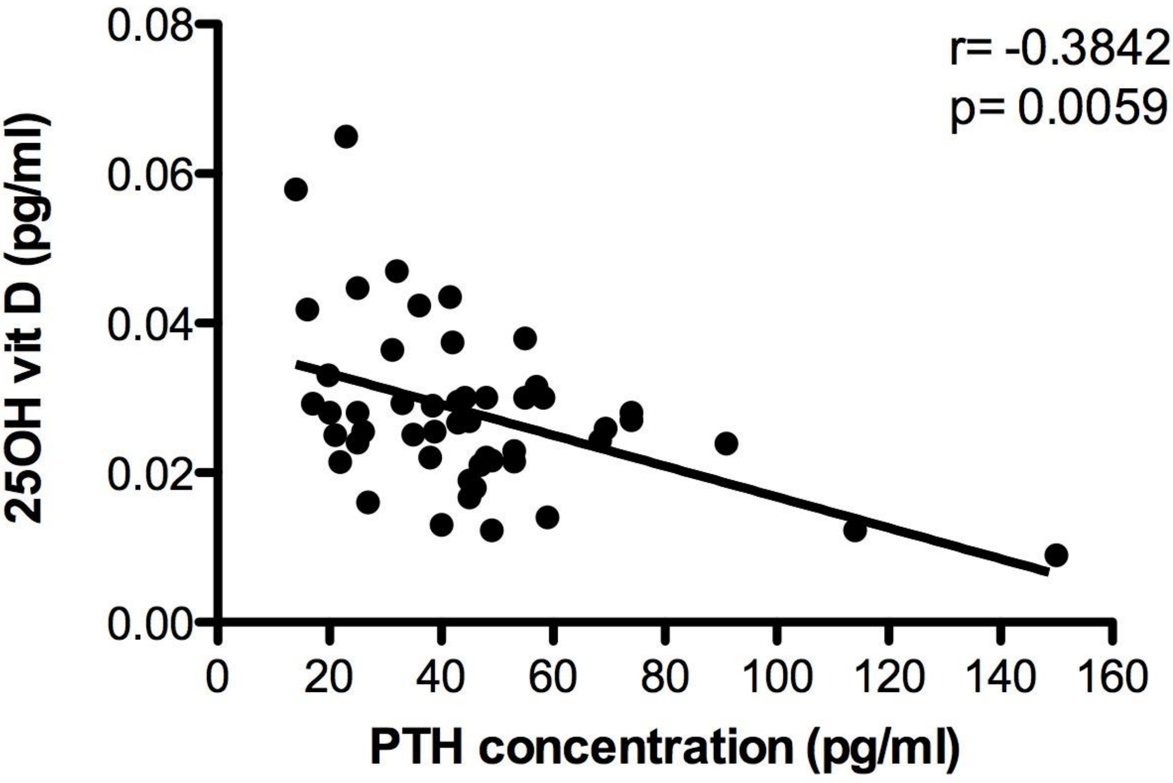

r= -0.3842
p= 0.0059
0.08
0.06
0.04
0.02
0.00
25OH vit D (pg/ml)
0
20
40
60
80
100
120
140
160
PTH concentration (pg/ml)
